# Improvement in Neuropathy Specific Quality of Life in Patients with Diabetes after Vitamin D Supplementation

**DOI:** 10.1155/2017/7928083

**Published:** 2017-12-28

**Authors:** Uazman Alam, Asher Fawwad, Fariha Shaheen, Bilal Tahir, Abdul Basit, Rayaz A. Malik

**Affiliations:** ^1^Department of Eye and Vision Sciences, Institute of Ageing and Chronic Disease, University of Liverpool and Aintree University Hospital NHS Foundation Trust, Liverpool, UK; ^2^Division of Diabetes, Endocrinology and Gastroenterology, School of Medical Sciences, University of Manchester, Manchester, UK; ^3^Baqai Institute of Diabetology and Endocrinology, Karachi, Pakistan; ^4^Baqai Medical University, Karachi, Pakistan; ^5^Weill Cornell Medicine-Qatar, Doha, Qatar; ^6^Institute of Cardiovascular Sciences, University of Manchester and Central Manchester Hospital Foundation Trust, Manchester, UK; ^7^Manchester Metropolitan University, Manchester, UK

## Abstract

**Objective:**

To assess the effect of vitamin D supplementation on neuropathy specific quality of life (NeuroQoL) in patients with painful diabetic neuropathy.

**Methods:**

This prospective, open label study was conducted between June 2012 and April 2013. Patients with symptomatic diabetic neuropathy were given a single dose of 600,000 IU intramuscular vitamin D, and NeuroQol was assessed at baseline and at five follow-up visits every 4 weeks.

**Results:**

Of 143 participants, 41.3% were vitamin D deficient (vitamin D < 20 ng/ml). Treatment with vitamin D resulted in a significant increase in 25(OH)D (*P* < 0.0001) and a significant improvement in the NeuroQoL subscale score for emotional distress (*P* = 0.04), with no significant change in the other NeuroQoL domains of painful symptoms and paresthesia, loss of temperature and touch sensation, unsteadiness, limitation in daily activities, and interpersonal problems. There was a significant reduction in patient perception about foot problems on QoL of “quite a lot” (*P* < 0.05) and “very much” (*P* < 0.0001) with a significant reduction in the baseline response of having a “poor” QoL from 5.2% to 0.7% (*P* < 0.0001) and an increase in the response of an “excellent QoL” from 1.5% to 7.4% (*P* < 0.0001).

**Conclusion:**

Vitamin D is effective in improving quality of life in patients with painful diabetic neuropathy.

## 1. Introduction

Painful diabetic neuropathy is a major complication of diabetes, characterised by pain, tingling, burning, and cramps in the feet and lower legs with a significant reduction in quality of life [1]. In our previous large population-based study in the UK, the prevalence of painful diabetic neuropathy was 21% in Europeans but 34% in South Asians [2]. The prevalence of painful diabetic neuropathy ranges from 50% in Turkey [3], 22–60% in the Middle East [4], and 69% in Pakistan [5]. These differences have been attributed to different populations from primary and secondary care, criteria for diagnosing painful neuropathy, and different patient demographics.

Vitamin D deficiency is more prevalent in South Asians [6], even in people living in South Asia including Pakistan [7], and in Pakistan, the prevalence of vitamin D deficiency ranges from 40% [8] to 83% [9]. Vitamin D deficiency itself has been associated particularly with diabetic peripheral neuropathy [10–12] rather than retinopathy or maculopathy [13, 14]. In a study conducted in Kuwait, vitamin D deficiency occurred in ~82% of patients with diabetic neuropathy compared to ~61% of patients without neuropathy (*P* < 0.05) [15]. A meta-analysis has confirmed this association and showed an odds ratio of ~2.9 (95% CI 1.84–4.50) in favour of diabetic neuropathy in patients with vitamin D deficiency [16]. Recently in China, vitamin D deficiency has also been shown to be an independent risk factor for diabetic peripheral neuropathy [17]. We have recently shown a significant reduction in the severity of painful diabetic neuropathy after treatment with vitamin D [18].

Patients with diabetes have a worse quality of life (QoL) compared to persons without diabetes [19], especially those with diabetic neuropathy [20]. Painful neuropathy reduces quality of life (QoL) which appears to be mediated through increased anxiety, depression, physical burden, emotional disorders, and limitation of mobility. Relief of neuropathic pain with pharmacological agents has been shown to improve QoL [21–23].

The current study assessed the effect of treatment with a single intramuscular injection of high dose vitamin D on quality of life in patients with painful diabetic neuropathy using the NeuroQoL questionnaire (a specific validated neuropathy QoL instrument).

## 2. Methods

This was a prospective, open label clinical trial in patients with painful diabetic neuropathy between June 2012 and April 2013 undertaken in the Baqai Institute of Diabetology and Endocrinology (BIDE), a tertiary care diabetes unit in Karachi, Pakistan. The study received ethics approval from the institutional review board (IRB) of BIDE. Study participants gave signed informed consent in keeping with the Declaration of Helsinki.

Patients with symptomatic diabetic neuropathy aged above 18 years and HbA1c ≤ 11% with no comorbidities were included in the study after obtaining informed consent. None of the patients were actively receiving vitamin D supplementation during recruitment. Participants received a single dose of intramuscular vitamin D₃ of 600,000 IU and were assessed at five follow-up visits every 4 weeks.

Height, weight (ZT-160 scales, WINCOM, Jiangsu, China), and blood pressure was assessed, and body mass index was calculated using the formula (weight in kg/height in m^2^).

The laboratory used internal quality controls for the measurement of 25(OH)D. Serum 25(OH)D was measured using the immunoenzymetric assay, based on a solid phase enzyme-linked immunosorbent assay (ELISA) performed on microtiter plates and was performed exactly as per the manufacturer's instructions. The cross reactivity for vitamin D2 (of the assay) as per manufacturer's assertion was 100% (relative to vitamin D3), and the assay has excellent correlation to existing globally recognized assays, in combination with good sensitivity and precision (EP17-A Protocols for Determination of Limits of Detection and Limits of Quantitation; approved guideline, standard published by Clinical and Laboratory Standards Institute). The linear range of the assay is 7.7 ng/ml to 122.9 ng/ml. Inter- and intra-assay variation of the in-house control was 2.5% and 9.2%, respectively.

Deficiency was defined as a 25(OH)D < 20 ng/ml, insufficiency as 25(OH)D values 20–30 ng/ml, and adequacy as a 25(OH)D ≥ 30 ng/ml. These cutoffs are based on the Endocrine Society Clinical Practice Guideline, 2011.

## 3. Neuropathy Specific Quality of Life Questionnaire (NeuroQol)

The NeuroQoL is a specific validated neuropathy and foot ulcer QoL instrument [24, 25] which assesses diabetic neuropathy-related physical and emotional problems affecting daily life and well-being. Each question has a Likert scale of 1 to 5 for frequency of symptoms where 1 represents “never” and 5 represents “all the time.” 27 questions were divided into six subscales, that is, painful symptoms and paresthesia; reduction or loss of ability to feel temperature and/or objects with the feet; unsteadiness while standing/walking; limitation in daily activities; interpersonal problems, for example, physical/emotional dependence on others; and emotional distress. Six questions are related to the patient's perception of symptoms affecting QoL, and two separate questions assess the overall impact of neuropathy on QoL [24].

## 4. Statistical Analysis

**Figure 1:**
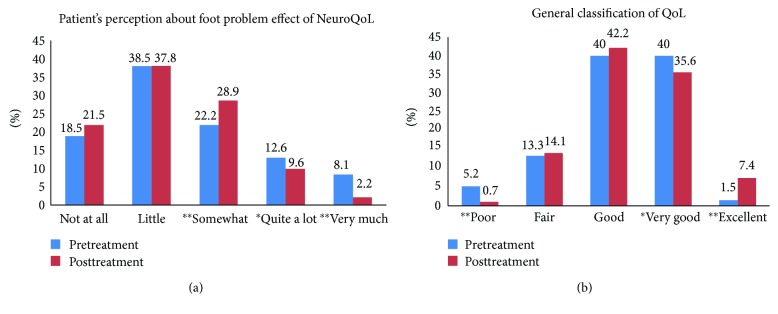
(a) Comparison of baseline and posttreatment patient's perception on QoL. (b) Comparison of baseline and post treatment general classification on QoL. ^∗^*P* < 0.05, ^∗∗^*P* < 0.0001.

All demographic, biochemical, and NeuroQol data were analysed using StatsDirect, Cheshire, UK. The data are presented as mean ± standard deviation (SD) and median with interquartile range where appropriate. All participants enrolled into the study were included in the analyses with missing data handled by using the Last Observation Carried Forward (LOCF). Student's *t*-test or nonparametric counterpart and chi-square test were used for analysis of anthropometric, metabolic parameters and patient's perception and general classification of QoL (Table 1, Figures 1(a) and 1(b)). The nonparametric Kruskall-Walis test was utilized with post hoc analyses (Conover-Iman) to test differences in NeuroQoL subscale scores from visit 1 to visit 5. Appropriate statistical tests were employed for both parametric and nonparametric data. Overall *P* value for multiple comparison tests were kept at 0.05. A *P* value of ≤0.05 was considered statistically significant.

## 5. Results

Table 2 summarises the anthropometric and demographic characteristics of the participants. 41% were males, 4.9% had type 1 diabetes, and 95.1% had type 2 diabetes. The age of participants was 52.3 ± 11.5 years, duration of diabetes was 12.1 ± 7.6 years, body mass index (BMI) was 29.7 ± 5.8 kg/m^2^, and HbA1c was 70.2 ± 16.4 mmol/mol. 40% had vitamin D deficiency (vitamin D < 20 ng/ml), 17% had insufficiency (vitamin D 20–30 ng/ml), and 43% had sufficient (vitamin D > 30 ng/ml) levels. There were no differences in anthropometric, clinical, and biochemical variables between patients with deficient, insufficient, and sufficient levels of vitamin D.

## 6. Effect of Treatment with Vitamin D (Table 1)

Treatment with vitamin D resulted in a significant increase in 25(OH)D (*P* < 0.0001), calcium (*P* = 0.009), and HDL (*P* = 0.03) and a reduction in HbA1c (*P* = 0.02) (Table 1). There was a significant improvement in the NeuroQoL subscale score for emotional distress by visit 3 (*P* = 0.04) which was maintained to the end of the study (Table 3). There were no significant changes in the other NeuroQoL domains of painful symptoms and paresthesia, loss of temperature and touch sensation, unsteadiness, limitation in daily activities, interpersonal problems, reduced QoL, and total QoL (Table 3).

After stratification of participants into those with sufficient (≥30 ng/ml, *n* = 80) and deficient (<30 ng/ml, *n* = 63) levels of vitamin D at baseline, we assessed the response to vitamin D in the NeuroQoL subscale for emotional distress (Table 4). Emotional distress improved significantly only in subjects with a vitamin D < 30 ng/ml (*P* = 0.04), whilst those with a baseline vitamin D status ≥30 ng/ml showed no significant change (Table 4). There was no change in any other domain from visit 1 to visit 5 when stratifying subjects based on sufficient (≥30 ng/ml) and deficient (<30 ng/ml) levels of vitamin D at baseline. There was also no correlation of baseline vitamin D with individual domains of the NeuroQoL using Kendall's rank correlation.

There was a significant reduction in the patient responses of “quite a lot” (*P* < 0.05) and “very much” (*P* < 0.0001) on patient perception about foot problem effect on QoL (Figure 1(a)). There was a significant reduction in the baseline response of having a “poor” QoL from 5.2% to 0.7% (*P* < 0.0001) and an increase in the response of an “excellent QoL” from 1.5% to 7.4% (*P* < 0.0001) (Figure 1(b)).

## 7. Loss to Follow-Up

Seven participants did not complete at visit 5, two participants at visit 4, one participant at visit 3, and one participant at visit 2. All participants enrolled into the study were included in subsequent analyses with LOCF for NeuroQol analyses.

## 8. Discussion

Diabetes and painful diabetic neuropathy are associated with reduced quality of life [20, 26–28]. Indeed, in a recent study the Norfolk, Qol-DN score was administered to 21,261 patients and demonstrated a 3-fold reduction in QoL in those with diabetic neuropathy [29]. Neuro-QoL is a validated tool for assessing neuropathy specific quality of life (QoL) in patients with diabetes [24] and has been employed to show an association between diabetic neuropathy and depressive symptoms [30]. The main findings of this study are that administration of a single high-dose treatment with vitamin D showed a significant improvement in specific NeuroQol subscale of emotional distress, improvements in the self-classification of QoL with fewer subjects classifying their QoL as poor, and “foot problems” causing less perceived reduced QoL. Pharmacological interventions for painful diabetic neuropathy may [28, 31] or may not [32] show an improvement in QoL due to significant side effects. Interestingly, a recent analysis of the COMBO-DN study has shown that treatment with duloxetine was of most benefit in patients without low mood [33].

A previous study has shown an improvement in pain, sleep, and quality of life after treatment with vitamin D in subjects with chronic pain [34]. We and others have demonstrated an improvement in pain-related symptom scores in diabetic patients with painful neuropathy after treatment with vitamin D [18, 35]. NeuroQoL is a neuropathy-specific QoL tool for the assessment and follow-up of therapeutic interventions in clinical trials of painful diabetic neuropathy. The SF-12 questionnaire has shown a profound effect on the physical and mental components of QoL in diabetic neuropathy [36]. However, the NeuroQoL physical symptom and psychosocial functioning scales shows stronger associations than the SF-12 with the clinical indicators of neuropathic severity, by mediating more fully the relationship of neuropathy to overall quality of life and explaining additional variance beyond that accounted for by the SF-12 measures [24].

In the present study, treatment with vitamin D resulted in an improvement in emotional distress after ~12 weeks, especially in those with low levels of vitamin D, suggesting a biological basis for this response [37]. Furthermore, patient perception of the general and more specific effect of foot problems on QoL was improved, suggesting that even small improvements in objective measures of neuropathic pain may improve QoL. Of course, even modest improvements in pain can produce clinically meaningful changes in function and status [38]. Whilst we [16] and others [26] have previously shown an improvement in the severity of painful neuropathic symptoms, in the present study, the subscale for positive and negative symptoms did not change, possibly because they evaluated the frequency rather than severity of these symptoms. Indeed, in a recent placebo-controlled study, treatment with vitamin D was associated with an improvement in positive neuropathic symptoms with no effect on neuropathic deficits or nerve conduction [10]. In relation to a lack of benefit on unsteadiness, we have recently shown no association between vitamin D deficiency and lower limb muscle strength or volume [39].

Increasing evidence suggests that an adequate intake of vitamin D should be encouraged particularly in populations at risk of vitamin D deficiency [10–12]. In the present study, we have shown an improvement in vitamin D levels as well as HbA1c and HDL [40], with an impact on neuropathy specific QoL.

This is an open label intervention study in a population attending a secondary care setting and therefore cannot be generalised to the whole diabetic population. We also cannot exclude a placebo response particularly as subjects received regular follow-up after a known active intervention, although the improvement was observed several weeks after treatment had commenced. We believe that the data warrant a larger double-blind placebo-controlled clinical trial with vitamin D to assess the benefits on painful neuropathic symptoms and QoL.

## 9. Conclusion

A significantly improved neuropathy-specific quality of life is observed following a single high-dose intramuscular treatment with vitamin D3 in patients with painful diabetic neuropathy, particularly those with vitamin D deficiency.

## Figures and Tables

**Table 1:** Baseline characteristics of patients.

Baseline characteristics	Total
*n*	143
Age (years)	52.3 ± 11.50
Male gender, *n* (%)	59 (41%)
Type 1	7 (4.9%)
Type 2	136 (95.1%)
Duration of diabetes (years)	12.1 ± 7.60
Body mass index (kg/m^2^)	29.7 ± 5.8
Systolic BP (mm/Hg)	126 ± 16
Diastolic BP (mm/Hg)	80 ± 9
Serum creatinine (mg/dl)	0.98 ± 0.30
Calcium (mg/dl)	8.7 ± 0.6
Serum cholesterol (mg/dl)	169 ± 41
Triglyceride (mg/dl)	134 ± 80
High-density lipoprotein (mg/dl)	39 ± 10
Low-density lipoprotein (mg/dl)	106 ± 34
HbA1c (%)	8.6 ± 1.5
HbA1c (mmol/mol)	70.2 ± 16.4
25(OH)D (ng/ml)	31.7 ± 23.2
Deficient (<20 ng/ml)	58 (40.6%)
Insufficient (20–30 ng/ml)	24 (16.8%)
Sufficient (>30 ng/ml)	61 (42.7%)

Data are presented as *n* (%) or mean ± SD.

**Table 2:** Baseline and end of trial anthropometric and metabolic parameters after administration of vitamin D.

Parameters (*n* = 143)	Baseline	Week 20	*P* value
Body mass index (kg/m^2^)	29.7 ± 5.8	30.2 ± 7.1	NS
Systolic BP (mmHg)	126 ± 16	123 ± 15	NS
Diastolic BP (mmHg)	80 ± 9	78 ± 7	NS
HbA1c (%)	8.6 ± 1.5	8.2 ± 1.5	0.02
HbA1c (mmol/mol)	70.2 ± 16.4	66.1 ± 16.6	
Creatinine (mg/dl)	0.98 ± 0.3	1.01 ± 0.4	NS
Calcium (mg/dl)	8.7 ± 0.6	8.9 ± 1.0	0.009
Serum cholesterol (mg/dl)	169 ± 41	162 ± 43	NS
Triglycerides (mg/dl)	134 ± 80	136 ± 78	NS
High-density lipoprotein (mg/dl)	39 ± 10	43 ± 11	0.03
Low-density lipoprotein (mg/dl)	106 ± 34	100 ± 34	NS
25(OH)D (ng/ml)	31.7 ± 23.2	46.2 ± 10.2	<0.0001

Data are presented as mean ± SD.

**Table 3:** NeuroQoL measures from baseline to final visit.

NeuroQoL measures	Baseline	V2	V3	V4	V5
Pain	11.7 ± 4.1	11.3 ± 4.7	11.5 ± 4.4	11.3 ± 4.3	11.4 ± 3.9
(−/35)	11 (9–14)	10 (8–13)	10 (8–14)	10 (8–14)	11 (9–13)
Loss reduction	4.9 ± 2.4	4.8 ± 2.4	4.8 ± 2.6	4.7 ± 2.6	4.6 ± 2.2
(−/15)	4 (3–6)	4 (3–6)	4 (3–6)	4 (3–6)	4 (3–5)
Diffuse sensory-motor symptoms	6.1 ± 3.6	6.1 ± 3.6	6.1 ± 3.5	6.1 ± 3.4	6.5 ± 3.0
(−/15)	5 (3–7)	5 (3–8)	5 (3–7)	5 (3–7)	6 (4–8)
Limitations	4.5 ± 2.5	4.6 ± 2.5	4.2 ± 1.9	4.2 ± 2.0	4.1 ± 2.0
(−/15)	3 (3–5)	3 (3–6)	3 (3–5)	3 (3–5)	3 (3–5)
Interpersonal	6.3 ± 3.9	6.3 ± 4.1	5.6 ± 3.1	5.9 ± 3.5	5.6 ± 3.1
(−/20)	4 (4–7)	4 (4–7)	4 (4–7)	4 (4–7)	4 (4–7)
Emotional distress	13.4 ± 8.1	12.1 ± 7.5	**11.0 ± 6.4**	**11.0 ± 6.0**	**10.8 ± 5.4**
(−/35)	9 (7–20)	7 (7–17)	**7 (7–13)** ^∗^	**7 (7–14)** ^∗^	**7 (7–15)** ^∗∗^
R QoL	12.0 ± 6.3	11.4 ± 6.2	11.2 ± 5.4	11.5 ± 5.2	11.2 ± 4.7
(−/30)	10 (7–16)	9 (7–15)	9 (7–14)	10 (7–14)	10 (8–13)
Total QoL	64.7 ± 24.3	62.3 ± 24.9	60.1 ± 21.8	60.2 ± 21.7	59.8 ± 18.2
(−/135)	57 (45–78)	54 (44–71)	53 (44–70)	54 (43–71)	55 (45–70)

Data are presented as mean ± SD and median (IQR). ^∗^*P* = 0.02 and ^∗∗^*P* = 0.03.

**Table 4:** Response in emotional distress stratified according to baseline vitamin D status (<30 ng/ml and ≥30 ng/ml).

NeuroQoL measures	Baseline	V2	V3	V4	V5
Emotional distress					
25(OH)D < 30 ng/ml	13.5 ± 8.2	11.9 ± 7.7	10.7 ± 6.4	**10.3 ± 5.3**	**10.4 ± 5.4**
*n* = 80 (−/35)	8.5 (7–21)	7 (7–16)	7 (7–11)	**7 (7–11.5)** ^∗^	**7 (7–14)** ^∗^
Emotional distress					
25(OH)D ≥ 30 ng/ml	13.4 ± 8.1	12.3 ± 7.2	11.4 ± 6.5	11.8 ± 6.8	11.3 ± 5.4
*n* = 63 (−/35)	10 (7–20)	7 (7–18)	7 (7–15)	7 (7–16)	7 (7–15)

Data are presented as mean ± SD and median (IQR), ^∗^*P* = 0.04.

## Additional Points

*Key Message.* Vitamin D may play an important role in the treatment of painful diabetic neuropathy in relation to quality of life. A single high-dose intramuscular injection of vitamin D3 provides significant improvements in neuropathy-specific aspects of the quality of life in patients with diabetic neuropathy. *Research Questions.* Are the quality of life improvements maintained over a longer period time? Through which central mechanism(s) does vitamin D cause improvement in quality of life? Are the same findings replicated in a randomised placebo controlled trial? *Guarantor of Data and Manuscript.* Asher Fawwad is the guarantor of this work and, as such, had full access to all the data in the study and takes responsibility for the integrity of the data and the accuracy of the data analysis.
